# Needlestick and sharps injuries among housekeeping workers in hospitals of Shiraz, Iran

**DOI:** 10.1186/1756-0500-5-276

**Published:** 2012-06-07

**Authors:** Parvin Lakbala, Farbood Ebadi Azar, Hajeb Kamali

**Affiliations:** 1 Medical Record and Health Information Technology, Hormozgan University of Medical Science, Bandar Abbas, 79168319, Iran; 2 School of Health, Tehran University of Medical Sciences, Tehran, Islamic Republic of Iran; 3 Obstetrics and Gynecology, North Bristol NHS, Becks pool Road, Frenchay Bristol, Bs161JE, United Kingdom

**Keywords:** Needlestick and sharps injuries, Biomedical waste, Blood-borne pathogens, Housekeeping work

## Abstract

**Background:**

Needlestick and sharps injuries (NSSIs) are one of the major risk factors for blood-borne infections (BBPs) at healthcare facilities. This study examines the current prevalence of NSSIs among housekeeping workers engaged in the handling and disposal of biomedical waste (BMW) at government and private hospitals in Shiraz, Iran, and furthermore, explores strategies for preventing these injuries.

**Findings:**

Using a cross-sectional study design, NSSI's and associated protective measures for housekeeping workers throughout hospitals in Shiraz were evaluated from 2009 onwards. Using a questionnaire, data was collected for 92 workers who had engaged directly with BMW. Data was analyzed using Chi-square, student t-test and where appropriate, SPSS version 12. 90.2 % of housekeeping workers were warned of the dangers associated with waste, 87.5 % in government and 93.2 % in private hospitals (P = 0.0444). 83.7 % had attended educational programs on biomedical waste (BMW) management and injury prevention at their hospital in the preceding year. 16.3 % had not been trained in biomedical waste management (P = 0.0379) and 88.9 % had a sufficient supply of safety wear.

**Conclusions:**

NSSIs are a common risk factor for infection among health care workers within hospitals in Iran. For the effective prevention of these injuries, health boards and hospital trusts need to formulate strategies to improve the working conditions of health care workers, discourage the excessive use of injections, and increase their adherence to universal precautions.

## Findings

Needlestick injuries are an important and common occupational injury amongst healthcare workers and have a significant impact on the morbidity and mortality of these workers through the transmission of BBP [1,2]. Biomedical or Healthcare waste is a term used for all waste that arises in healthcare establishments. Syringes used in hospital are sharps, one of the types of risk waste [3]. The highest risk groups are doctors, nurses, healthcare auxiliaries, hospital maintenance personnel and patients in healthcare establishments [4]. Among the 35 million health care workers worldwide, three million experience needlestick and sharps injuries (NSSIs) every year [5].

More than 100,000 NSSI are reported in UK hospitals annually[6], posing a considerable risk for the transmission of more than 20 kinds of blood-borne pathogens, including hepatitis B virus (HBV), hepatitis C virus (HCV), and human immunodeficiency virus (HIV) [2]. The World Health Organization has estimated that exposure to sharps in the workplace accounts for 40 % of infections with HBV and HCV and 2-3 % of HIV infections among health care workers [7]. These occupationally related infections have implications on the psychological and physical wellbeing of those they affect but also have a socio-economic impact and are a financial burden for accident and insurance institutions. In recognition of these consequences, prevention measures have been the focus of hazard reduction for health care professionals [8].

The major activities causing needle stick injuries are administering injections, blood sampling, recapping needles, needles disposal, handling non-biological waste and dirty linen (downstream injuries) and during the transfer of bodily fluid from a syringe to a specimen container (such as a vacuum tube) [9]. Safety practice has a serious role in safety and health maintenance of health care workers [10].

The overall rate of transmission of HBV among susceptible HCW without post-exposure prophylaxis or up to date vaccination has been estimated as between 6 % and 30 % [11] although estimated rates of HBV infection among HCW in the U.S.A have declined since the 1980s by around 95 % [12]. Potential reasons for this decline include the licensing and increasing use of an effective vaccine and changes in work practices, such as the adoption of universal precautions [11]. However, evidence from the US suggests that more than half of all sharps-related injuries are not reported [13]. Poor reporting of sharps-related injuries reveals a failure to appreciate the potential consequences of such injuries [14].

The purpose of this study is to evaluate needle stick and sharps injury among housekeeping workers involved in hospital waste handling and disposal. Alongside this, we also aim to assess awareness of the implications on public and environmental health relating to improper management of health care waste amongst this group as well as the use of protective and precautionary measures against NSSI.

## Methods

A survey was conducted in 2009 of 92 housekeeping workers in 9 hospitals, made up of 4 government and 5 private hospitals in the city of Shiraz, Iran. The survey probed into the practices adopted for the handling of needlesticks and sharps and the management of NSSIs. Randomization was used to select 9 out of 33 hospitals in Shiraz. These hospitals have a total of 1503 beds and accept 3666 outpatients and 1019 inpatients per day. A questionnaire was distributed amongst the selected hospitals and this was independently completed by the cohort of HCW. Out of 486 housekeeping workers in the selected hospitals, 92 workers were identified as being at risk of NSSI using the inclusion criteria of direct engagement in in the collection, handling and disposal of bio-medical waste including the handling of sharps contaminated with blood. The questionnaire included 31 questions and was in the format of either multiple choice questions or free text. The questions revolved around:

· Workers educational and training conditions

· Awareness of the importance and hazards associated with medical waste

· Monitoring and training BMW management systems

· Vaccination of workers

· Workers reaction to NSSI

· Protective clothing

· Occurrence of sharps injuries in the past 12 months

· Workers beliefs about safety

· Workers knowledge of precautions

· Hospital placed precautionary measures against NSSIs

· The situation causing the NSSI

The characteristics of the occupational exposures were also asked, including the use of protective measures, immunization status and prophylactic management following the exposure. Two experts reviewed the initial questionnaire prior to a pilot sample being tested on25 housekeeping workers.

To evaluate the questionnaire’s reliability, 10 % of participants were retested again 2 weeks later. The reliability of the questionnaire was confirmed by Cronbach's alpha calculation (α = 0.87).

We obtained ethical approval from the Shiraz Medical Science University for data collection about biomedical waste management and workers knowledge and awareness about dangers associated with handling waste.

The answers regarding workers awareness were grouped into 'positive' and 'negative' responses.ve answers. The data was then coded, tabulated and analyzed using SPSS version 12, Chi-square test, T- test and a p < 0.05 was considered statistically significant.

## Results

83.7 % (77/92) of housekeeping workers had knowledge of the BMW system in their hospital. 79.2 % (38/48) of Government hospital workers had knowledge of BMW management systems compared with 83.7 % (39/44) of Private hospital workers. Government hospital workers knowledge of BMW management (mean = 9.94) is significantly less than the Private hospitals workers (mean = 10.82), (t = 2.353, P = 0.021). 90.2 % (83/92) of housekeeping workers were aware of the dangers associated with waste, 87.5 %( 42/48) in government and 93/2 %( 41/44) in private hospitals. The data suggests that workers within the private sector appear to have superior training and education on BMW and associated hazards (Table 1).

**Table 1:** Housekeeping workers awareness with BMW

**Workers awareness**	**Government**		**Private**		**Total**	
	Yes	No	Yes	No	Yes	No
**Awareness of dangers associated with waste**	42(87.5)	6(12.5)	41(93.2)	3(6.8)	83(90.2)	9(9.8)
**Awareness of BMW management**	38(79.2)	10(20.8)	39(88.6)	5(11.4)	77(83.7)	15(16.3)
**Training on BMW management**	36(75.0)	12(25.0)	41(93.2)	3(06.8)	77(83.7)	15(16.3)
**Vaccination against tetanus &hepatitis**	44(91.7)	4(8.3)	41(93.2)	3(6.8)	83(92.4)	7(7.6)
**Workers satisfaction with the present BMW system**	35(72.9)	13(27.1)	40(90.9)	4(9.1)	75(81.5)	17(18.5)

Figures in parentheses are percentages.

In Table 2, precautions taken by the hospital workers in their BMW management efforts are shown in both the Government and the Private hospitals. The precautionary activities include such things as using protective clothing and equipment while handling the biomedical wastes. The incidence of NSSIs during one year prior to the survey among workers was 22.8 % (21/92). The incidence was 31.3(15/48) for Government hospitals (educational hospitals), and 13.6 % (6/44) for private hospitals. A statistically significant difference was found between the workers of the Private and Government hospitals in relation to NSSI (p = 0.0444). As regards their knowledge of NSSI's, 83/7 % of 92 respondents had attended educational programs on biomedical waste management and injury prevention at their hospital in the preceding year and 16.3 % (15/92) were not trained in biomedical waste management (P = 0.0379) with 88.9 % having access to safety wear.

**Table 2:** Precautions taken by workers of BMW management

**Precautions workers took**	**Government**	**Private**	**Total**
Put segrated waste in special waste rooms	6 (12.5)	2 (4.5)	8 (8.7)
Wear gloves and masks	6 (12.5)	12 (27.3)	18 (19.6)
Wear gloves and masks and put segregated wastes in trolleys for transfer	24 (50.0)	19(43.2)	4(46.7)
Wear gloves, masks and aprons	1 (2.1)	2 (4.5)	3 (3.3)
Regular washing of buckets and wearing gloves and mask	3 (6.3)	3 (6.8)	6 (6.5)
Wear hand gloves and boots	2 (4.2)	-	2 (2.2)
Put segregated waste in trolleys and wear masks, gloves and aprons	-	3 (6.8)	3 (3.3)
Wear protective clothing and careful handling	5 (10.4)	3 (6.8)	8 (8.7)
All	1 (2.1)	-	1 (1.1)
Total	48(100.0)	4(100.0)	9(100.0)

Figures in parentheses are percentages.

The training programs were conducted by the environmental health specialists and the infection control committees that work within the hospitals.

The data implies that workers in Government hospitals incur more needle stick injuries than those in the Private hospitals. This is most likely a result of a lack of worker awareness and knowledge, partially due to poor training and education programs.

When questioned about reporting NSSI's, procedures fed back by workers included reporting to supervisors, washing the injury site with soap and antiseptic, consulting with a doctor (and undergoing the appropriate investigations), applying pressure to the injury site and receiving post-exposure vaccines. These procedures are often done in combination rather than individually. Of the 92 housekeeping workers, 8.7 % (8/92) reported to supervisors, 1.1 %( 1/92) washed the wound with water, soap or antiseptics, 4.3 %( 4/92) consulted a doctor and provided blood samples for HIV or HBV in laboratory. 38/0 % (35/90) carried out all the previously quoted procedures, 34.8 % (32/92) had done all measures above and applied pressure to the wound site and 9.8 % (9/92) had done all of the above and also received a vaccine. Only 3.39 %( 3/92) underwent all the stated procedures following NSSI. Procedures workers adopted in case of an injury are shown in Table 3.

**Table 3:** Procedures workers adopted in case of an injury

**List of precautions workers took**	**Government**	**Private**	**Total**
Report to supervisors	4 (8.3)	4 (9.1)	8 (8.7)
Wash injury site with soap and betadine	1 (2.1)	-	1 (1.1)
Visit doctor and do some tests	4 (8.3)	-	4 (4.3)
Report to superisors, visit doctors and do some tests	21 (43.8)	14 (31.8)	35 (38.0)
Report to superisors, visit doctors, do some tests and press injury site	7 (14.6)	25 (56.8)	32 (34.8)
Report to superisors, visit doctors, do some tests and inject vaccine	8 (16.7)	1 (2.3)	9 (9.8)
All	3 (6.3)	-	3 (3.39)
Total	48 (100)	44 (100)	92 (100)

Figures in parentheses are percentages.

Hospital workers should be vaccinated against Tetanus and Hepatitis B and of our cohort of 92, 92.4 %( 85/92) had received both.

A number of limitations to the study can be identified. Firstly, the study concentrated on issues relating to NSSI's due to biomedical waste management only. The study can be further expanded by focusing on issues related to Hygiene, Environment, Sanitation, Health and Safety. The study was conducted on housekeeping workers who were directly engaged in collecting, handling and disposal of BMW in 9 hospitals within Shiraz. More representative and generalizable results would have been obtained if the sample was extracted from a larger cohort of workers.

## Discussions

Needlestick injuries in HCWs are an important and significant occupational hazard and can potentially lead to infections with blood-borne pathogens such as HBV, HCV, or HIV [15-18]. It is important to increase awareness around the prevalence and dangers of NSSI have in order to find alternative ways in which to reduce the incidence.

The present study describes the prevalence of NSSI amongst housekeeping workers involved in hospital BMW handling and disposal but also highlights the level of knowledge of these workers with regards to the public and environmental health implications of improper management of BMW.

The survey was carried out through an anonymous questionnaire and it focused on injuries caused by contaminated sharps, including needles, lancets, and scalpels. Within hospitals of Germany, a specialist consultant in emergency medicine is allocated and dedicated to reporting and managing occupational incidents such as NSSI as well as administering and advising on post-exposure prophylaxis [19]. The data of a Japanese teaching hospital also show a poor reporting rate of NSSI incident [20]. According to NHS policy in the UK, it is compulsory when staff sustain a needle-stick injury to report the incident [21]. However, evidence from the US suggests that more than half of all sharps-related injuries are not reported [22]. In our study, the infection committee receives only minimal reporting on NSSI's and reporting is not considered compulsory. Poor reporting of sharps-related injuries reveals a failure to appreciate the potential consequences of such injuries [14].

There are different strategies to prevent infections due to sharps injuries, including training HCWs and a reduction in unnecessary invasive procedures. Vaccination is one of the best ways to protect HCWs from infection, but vaccination is only available for HBV and Tetanus. In the present study, the number of vaccinated workers was 92.4 %. This figure would suggest that a greater awareness of the requirement of the HBV vaccination is required [23]. Many sharps injuries happened before and during sharps disposal, but were also due to unsafe sharps disposal boxes. These incidents would not have occurred if a safety device was used and the needle safety mechanism had been activated. These findings were supported by the results of recent studies in the USA, Canada, Japan, UK, and Germany [24-28].

This study has demonstrated that the inadequate reporting of NSSIs to medical staff was a common occurrence amongst health care workers. As many as two third of the survey participants did not seek medical treatment after an injury, which is a similar finding to studies carried out elsewhere [8,29,30]. The most common reasons for this are insufficient knowledge and poor practices. The observed high level of under-reporting suggests that workers need education on prevention, especially focusing on the importance of reporting all NSSI's and the possibilities of prophylaxis after exposure to BBP [31-33]. In our study all workers reported that the most common cause of injuries from needles in housekeeping workers was improper handling of needles, the use of overly full sharps containers and misplaced needle left by students or HCW's, especially within teaching hospitals. This echoes Bilsk et al, which reported that the most common cause of injuries from needles in nurses was improper handling of syringes and needles after injections (removing a needle from a syringe or placing the needle in a full container for medical waste) [34].

This study showed that adherence to the universal precautionary recommendations was another important factor in the prevention of NSSI's, a finding which is in accord with previous studies [8,29,35]. Given that safer sharps devices or devices with a built-in safety feature are not yet widely available in Iran, strengthening education and training systems is thus essential. This can be achieved through attendance at seminars designed to enhance awareness of the standard precautions and protocols, knowledge of which seems to be still far from adequate in Iran. As post exposure prophylaxis has been shown to be effective after these injuries [36], a system should be introduced to ensure that all health care workers have knowledge of appropriate means through which they can seek medical treatment and other follow up care following NSSI. The introduction of a system for the computerized collection of information in work is also required.

Reducing the risk of NSSIs through improved occupational health standards and safety management systems would ultimately decrease the burden of disease on society from infections with blood-borne pathogens in Iran as universal precautions play an important role in minimizing and preventing exposure of health care workers to such pathogens [37]. There is also a need to develop strategies to promote the use of universal precautions which take into account behavior change and accrual of knowledge including its integration into practice. Housekeeping workers are at high risk of NSSIs and hence BBP exposure and we recommend that they would benefit from a targeted education program about protective strategies against blood-borne infection. Iran needs an obligatory training programme in healthcare precautions, requiring involvement of senior health staff in the development and implementation of policies and systems for monitoring the appropriate use of equipment as well as establishing a post exposure reporting system.

## Competing interests

The authors declare that they have no competing interests.

## Authors’ contributions

PL conducted the statistical analysis, interpreted the data, drafted the manuscript, and critically revised the manuscript for intellectual content. FE supported the data collection; critically revised the manuscript.HK critically revised the manuscript. All authors read and approved the final manuscript.
